# Attenuation law of concentrated stress under coal pillar of close coal seams and its application

**DOI:** 10.1038/s41598-022-26036-x

**Published:** 2022-12-16

**Authors:** Qingtao Kang, Fulian He, Shuaifeng Yin, Yang Yang

**Affiliations:** 1 School of Energy and Mining Engineering, China University of Mining and Technology (Beijing), Beijing, 100083 China; 2 School of Safety Engineering, North China Institute of Science and Technology, Sanhe, 065201 Hebei China

**Keywords:** Civil engineering, Geophysics

## Abstract

When mining the multiple coal seams in close proximity, the coal pillar left in the goaf causes stress concentration in the floor. The layout of mining roadway in lower short distance coal seam is affected by the propagation of concentrated stress caused by the upper coal pillar. To determine the reasonable distance of the roadway in the lower coal seam outside the coal pillar, the attenuation law of concentrated stress outside the coal pillar boundary has been studied through simulation model, theoretical analysis, and example analysis. The results show that the concentrated stress of coal pillar decreases with the distance from the coal pillar. At the coal pillar boundary position, the stress change rate reaches the maximum in the floor with different depths, and the stress decreases rapidly in the floor strata outside the coal pillar. Under the same stress condition, the roadway layout in strata at different depths is different. The joint formula of stress and stress change rate was deduced to determine the reasonable horizontal distance of roadway outside coal pillar. The results obtained by the numerical simulation fitting formula and the theoretical calculation formula are close to each other when calculating an engineering example. The roadway pressure appearance is not obvious in the experiment and physical simulation, which indicates that the theoretical formula can satisfy the requirement of engineering calculation. The method provides a reference for roadway location selection under similar conditions.

## Introduction

**Figure 1:**
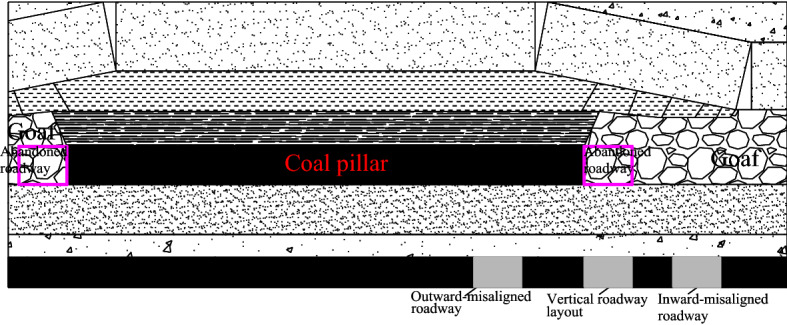
Three roadway location layout under coal pillar.

Close distance coal seam groups are widely distributed in China, such as Xishan mining area, Lvliang mining area, Datong mining area, Lianghuai mining area, etc^1–4^. Due to the small distance between the coal seams, the interaction is obvious in the process of close coal seam mining. In the mining process of close coal seams, the remaining coal pillars left over in the upper coal seam have a greater impact on the lower nearby roadway’s layout and its surrounding rock control mode. If the lower coal seam is within the influence range of the concentrated stress of the upper remaining coal pillar, the coal mining roadway located near the coal pillar can be roughly divided into three kinds of layout forms^1,5–7^, which are called outward-misaligned roadway layout, inward-misaligned roadway layout, and vertical roadway layout (Fig. 1). The outward-misaligned roadway is in the high stress area under the coal pillar. The inward-misaligned roadway is in the low-stress area outside the coal pillar. The vertical roadway is in the stress-decreasing area at the boundary of the coal pillar. At present, researchers reached an agreement that the roadway layout in close distance coal seam should avoid the high stress area under the coal pillar as far as possible^7–10^. In other words, the inward-misaligned roadway layout is well recognized as the better form in such circumstances.

**Figure 2:**
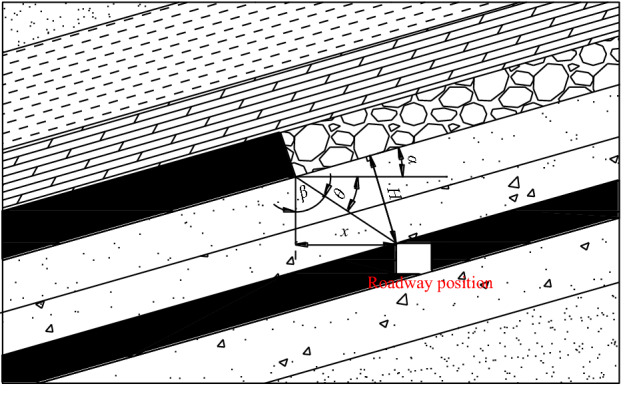
Calculation diagram of roadway location in the floor^10–14^.

There have been some research findings and engineering applications of inward-misaligned roadway layout under the coal pillar in the close coal seam. For example, some scholars used the formula (1)^11–15^ with the parameters in Fig. 2 to calculate the misaligned distance between the coal pillar in upper coal seam and the roadway, according to the stress propagation law^12^ in the coal pillar floor. This method has the following characteristics.

1. The formula calculation is based on the influence angle of the stress caused by coal pillar, which results that the roadway is arranged in the area where the stress is lower than the original in-situ stress. The influence angle refers to the angle between isoline of the original in-situ stress and the vertical line of the coal pillar boundary. However, this location is still in a stress-decreasing zone, and the stress in the roadway surrounding rock decreases gradually when reaching away from the coal pillar, which makes the stress in roadway surrounding rock unbalanced.
2. The value of the stress influence angle *β* under different conditions is different. Due to the large value range of the stress influence angle, it is difficult to select the stress influence angle of the coal pillar in practical engineering applications.
3. In the formula, the distance between the isoline of original rock stress and the boundary of the coal pillar is set to vary linearly with depth in defaults. Whereas, in reality there is a different scenario because the stress isoline of the original rock is not a straight line^12,16^. When the roadway is far away from the coal pillar, the calculation would be accurate.
  1 $$x \ge \frac{{{\text{Hsin}}\beta }}{{{\text{sin(}}\theta + \alpha ) }}$$

Significant research achievement on the distribution range of the stress reduction zone near the coal pillar boundary have been made in previous research ^4,8,16–24^. To identify the variables that affect stress redistribution in the strata underneath supercritical longwall panels, the Wilson’s equations were used to evaluate vertical stress [formula (2), (3)] in the underlying strata^20^. A fitting function about the relationship of the coal pillar width and the distance between the boundary of the coal pillar and the boundary of stress reduction zone was obtained according to the numerical simulation results in reference^22^. Some scholars confirmed the reasonable location of the roadway outside coal pillar by analyzing the stress change rate^4,8^. According to the problem of roadway layout under the coal pillar at close distance, some scholars proposed the "three index method"(concentrated stress ξ, lateral pressure coefficient λ, stress gradient δ) to describe the stress field in the field. Their results argue that the roadway should be preferentially arranged in the area with conditions of "0 ≤ ζ ≤ 1, 0.8 ≤ λ ≤ 1.4, 0 ≤ δ ≤ 1"^23,24^.

The equation for the vertical stress distribution in the yield zone^20^:

2 $$\sigma_{y} = k(p + p^{\prime})\left( {\frac{x + M/2}{{M/2}}} \right)^{k - 1}$$

The equation for the vertical stress in the elastic zone^20^:

3 $$_{{C = \frac{0.15H - M/2}{{(k - 1) + \sigma_{0} /q}}}}$$

At present, determination of the value of the stress change rate and the boundary position of the stress reduction zone in the coal pillar floor are mostly based on numerical simulation and stress fitting function to determine the value of the stress change rate and the boundary position of the stress reduction zone, and more detailed theoretical studies are absent. Based on the concentrated stress propagation law in the floor under the coal pillar, the present paper further analyzes the law of stress attenuation affected by the coal pillar, the reasonable position of the roadway with different depths under the coal pillar is determined, so as to provide a theoretical basis for similar projects.

## Numerical simulation study on the attenuation law of concentrated stress under coal pillar

### Explanation of numerical simulation

The simulation software UDEC was used for the numerical simulation. A Chinese Mine serves as the engineering background for the mining relationship between #4 and #5 coal seams and coal pillars. Numerical simulation was carried out to study the transfer law of concentrated stress generated by the residual coal pillar in #4 coal seam floor. The width of the remaining coal pillar of the #4 coal seam is 45 m, and the interval between the #5 coal layer under the coal pillar is 5~6.5 m. Brief information of coal and rock strata are shown in Table 1. The numerical model is 517 m wide and 68 m high. The Mohr–Coulomb criterion was adopted in the model as material failure criterion, and the joint contact adopted coulomb sliding model was adopted to describe joint contact.

**Table 1:** Physico-mechanical properties of the strata.

Lithology	Density (kg/m^3^)	Bulk modulus (GPa)	Shear modulus (GPa)	Cohesion (MPa)	Friction (°)	Tension (MPa)	Jkn (GPa)	Jks (GPa)	Jfric (°)
Sandstone	2700	28.5	12.1	25	23	4.5	2.9	1.4	16.2
Fine sandstone	2750	79.1	28.3	45	22.5	4.6	3.9	2.4	20.2
Sandy mudstone	2500	6.17	4.07	12	30	2.6	2.6	1.68	18.7
#2 coal seam	1400	1.32	0.6	1.2	15.3	0.7	1.8	1.5	16.1
Sandy mudstone	2500	6.17	4.07	12	30	2.6	2.6	1.68	18.7
#3 coal seam	1400	1.32	0.6	1.2	15.3	0.7	1.8	1.5	16.1
Siltstone	2700	7.5	4.5	15.6	26.5	3.8	2.4	1.5	26.3
Sandy mudstone	2500	6.17	4.07	12	30	1.6	2.6	1.68	18.7
#4 coal seam	1329	1.32	0.6	1.2	15.3	0.7	1.8	1.5	16.1
Siltstone	2700	7.5	4.5	15.6	26.5	3.83	2.4	1.5	26.3
Mudstone	2517	2.5	1.8	3.3	21.5	2.3	2.6	1.68	18.7
#5coal seam	1407	1.32	0.6	1.2	15.3	0.7	1.8	1.5	16.1
Fine sandstone	2750	79.1	28.3	45	22.5	8.26	3.9	2.4	20.2
Sandy mudstone	2500	6.17	4.07	12	30	2.6	1.6	1.68	18.7

According to the distance from the surface to the simulated rock layer at the top of the simulation model, a uniform distributed load of − 8.39 MPa, corresponding to a depth of 335 m, was used to represent the overburden weight in the actual stratum. Deformation at the bottom and sides of the model is fixed. In the model, the rock properties of different strata are shown in Table 1.

### Propagation and attenuation law of concentrated stress caused by the coal pillar in floor

**Figure 3:**
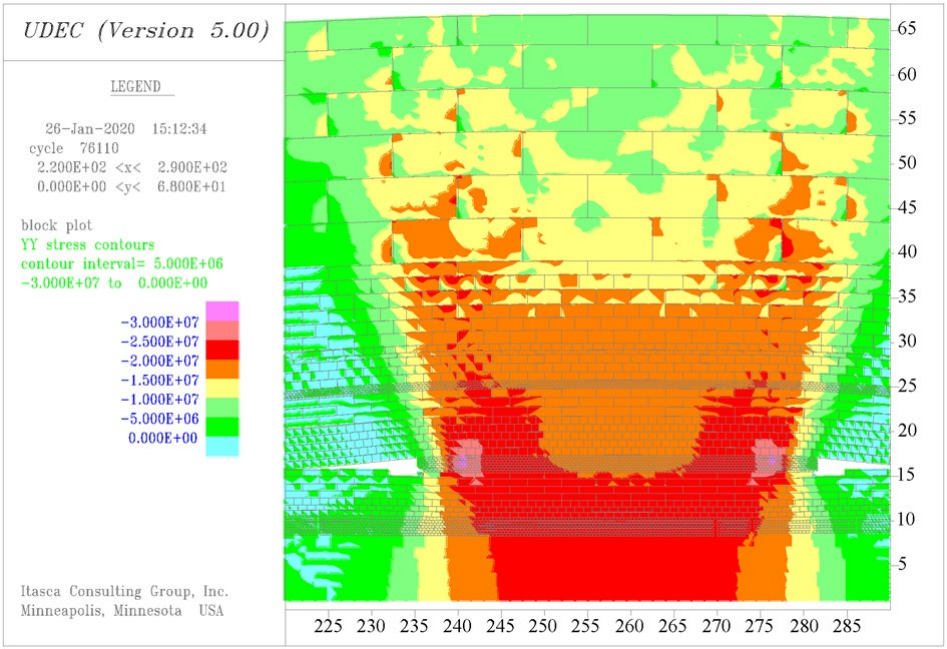
Stress distribution in the roof and floor of coal pillar.

According to the numerical simulation software calculation, the contour map of vertical stress in the roof and floor of the coal pillar of #4 coal seam is shown in Fig. 3. In the influence range of coal pillar concentrated stress, the purple region has the highest stress value, which is the peak area of the abutment pressure near the coal seam boundary. The red area is the higher stress area under the coal pillar. The stress in orange, yellow, and light green regions on both sides decreases successively, which is the boundary stress decreasing zone of the coal pillar. The green area is the low stress area outside the coal pillar.

As can be seen from Fig. 3, the width of the red high stress zone decreases with the distance from the coal pillar, and the widths of orange, yellow, light green, and green areas on both sides relatively increase, which shows that the influence of coal pillar on the stress concentration is gradually weakened, and the stress is gradually transferred to the two sides.

Three horizontal survey lines of pline1, pline2, and pline3 were set at the depth of 1 m, 5 m, and 10 m below the coal pillar, respectively, to record the stress distribution. Stress distribution along each line can be divided into high stress area, decreasing stress area, and low stress area.

**Figure 4:**
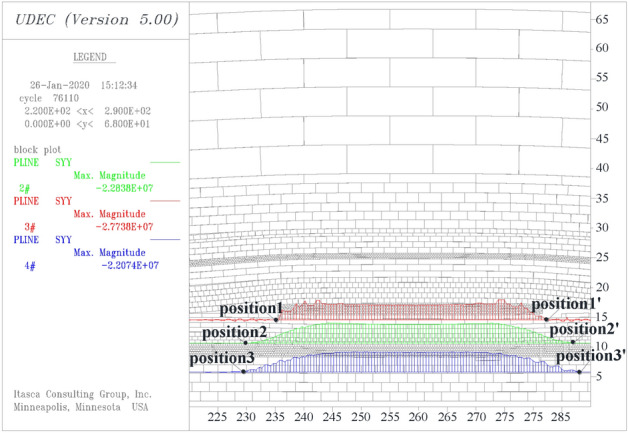
Stress distribution on different test lines.

As shown in Fig. 4, the range of the high-stress area decreases toward the center of the coal pillar as the depth increases, and the peak stress phenomenon becomes gradually insignificant as the depth increases. The locations of the stress decreasing area and the low stress area boundary have been marked as position1, position2, and position3 on the three survey lines (The stress change rate significantly reduces at these locations). The boundary position moves to both sides of the coal pillar with increasing depth, and the width of the stress reduction zone increases with the increase of depth.

### Selection of roadway location based on propagation attenuation law of coal pillar concentrated stress

The arrangement of the roadway under the coal pillar of the close coal seam should avoid the high stress area as much as possible, and the roadway should be arranged in the lower stress area so that the pressure of the surrounding rock of the roadway is small and easy to control. It should also be considered to avoid the vertical stress decline area to prevent the roof of the roadway from being subjected to greater shear stress. Therefore, the position where the vertical stress and its rate of change are both lower is a better layout position for the roadway.

**Figure 5:**
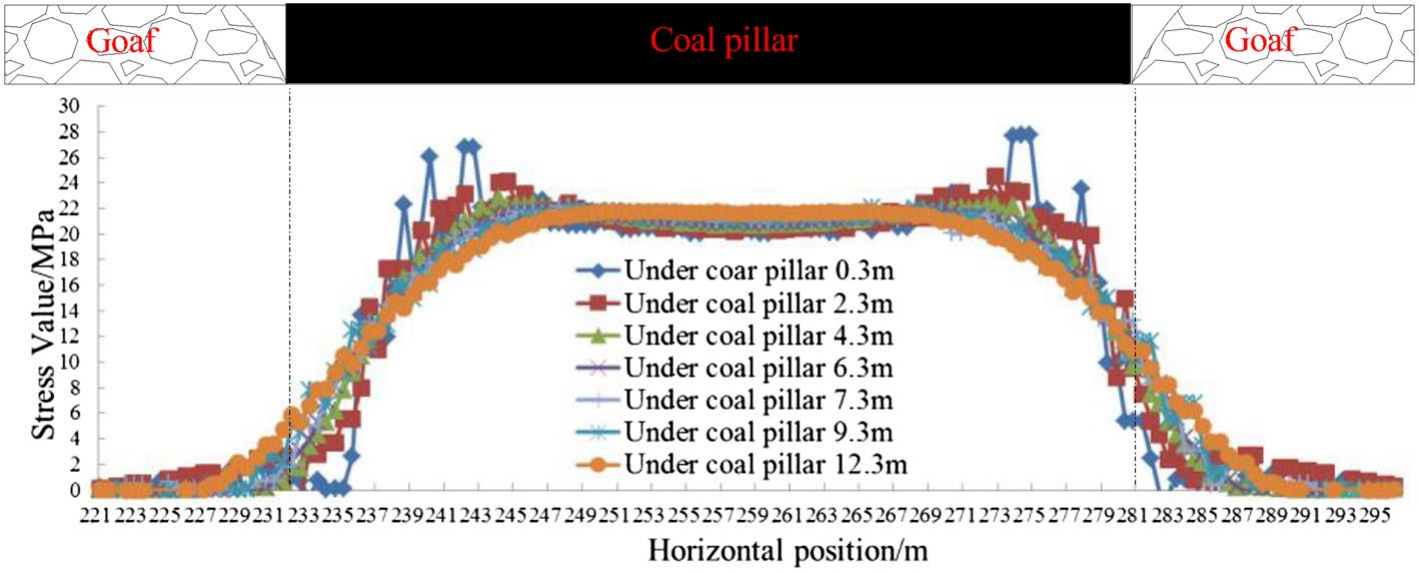
Stress change under coal pillar 0.3 m to 12.3 m.

For specific conditions, numerical methods can be used to analyze the stress distribution law, so as to determine the location where the stress and its change rate are low. According to the numerical results of the stress distribution in the coal strata under the coal pillar, the horizontal change curves of the vertical stress value in the floor at different depths under the coal pillar were drawn (Fig. 5). Six strata vertical stress measuring lines were respectively arranged under the coal pillar at 0.3, 2.3, 4.3, 6.3, 7.3, 9.3 and 12.3 m, with a measuring point spacing at 0.5 m. The stress curves of different measuring lines have certain fluctuations, but when they are getting far away from the coal pillar boundary, the stress value and the stress change rate are all reduced to very small values. Hereinafter, the position where the stress value first decreases to the small value is named the critical position of the stress reduction zone and the low stress zone.

According to the change law of the vertical stress in the horizontal direction, the horizontal distance between the critical position in the floor and the coal pillar boundary is different at varying depths. Therefore, the relationship between the critical position and the depth can be obtained by measuring the critical position data of the survey line at different depths.

**Figure 6:**
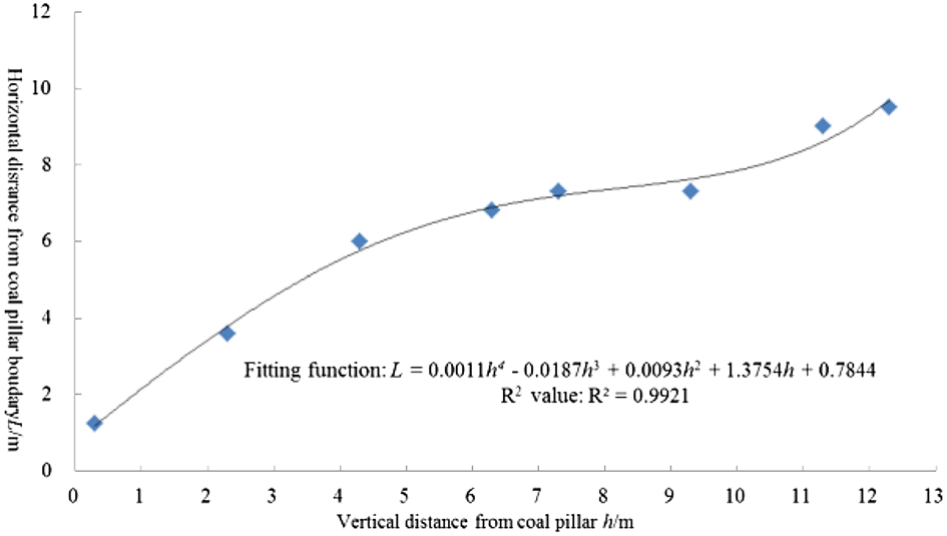
The relationship of the roadway horizontal distance and vertical distance from the coal pillar.

Through data analysis, it can be known that the depth of the floor at the critical position and the horizontal distance from the coal pillar boundary have a function relation of one variable. According to the numerical simulation results, the values were obtained and fitted to the unary quartic function formula (Fig. 6). The deviation of the fitted function data was relatively low.

According to the principle of selecting the optimal layout position of the roadway, the critical position obtained by fitting function can be used as the optimal position of coal pillar floor roadway. The fitting function and the coal seam interval show that the range of the stress decline zone and the optimal layout of the roadway under certain conditions can be obtained at the range of simulation measurement depth, but the universal law of stress change requires further theoretical analysis.

## Theoretical analysis of coal pillar concentrated stress propagation and attenuation

According to the distribution pattern of floor stress curve (Fig. 4) in numerical simulation and other literatures^12,25–28^, there are small crushing zones and corresponding stress fluctuations at the coal pillar boundary. Under the condition where the crushing zone widths of coal pillar are much smaller compared with the width of coal pillar, the stress fluctuation of coal pillar boundary has little effect on the floor. For the convenience of calculation, it is assumed that the concentrated force of coal pillar floor is uniform load and the floor strata are uniform elastic body.

**Figure 7:**
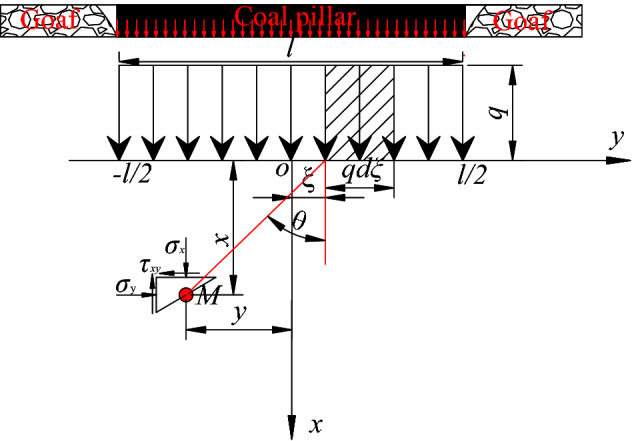
Mechanical calculation diagram of concentrated uniform stress acted to coal pillar floor.

The elastic mechanics theory^29,30^ provides a case of semi-infinite plane body with uniformly distributed load as shown in Fig. 7. The stress propagation formula in the floor rock under the coal pillar is as follows:

4 $$\sigma_{x} = \frac{q}{\pi }\left[ {\arctan \frac{{y - \frac{l}{2}}}{x} - \arctan \frac{{y + \frac{l}{2}}}{x} + \frac{{x\left( {y - \frac{l}{2}} \right)}}{{x^{2} + \left( {y - \frac{l}{2}} \right)^{2} }} - \frac{{x(y + \frac{l}{2})}}{{x^{2} + \left( {y + \frac{l}{2}} \right)^{2} }}} \right]$$

**Figure8:**
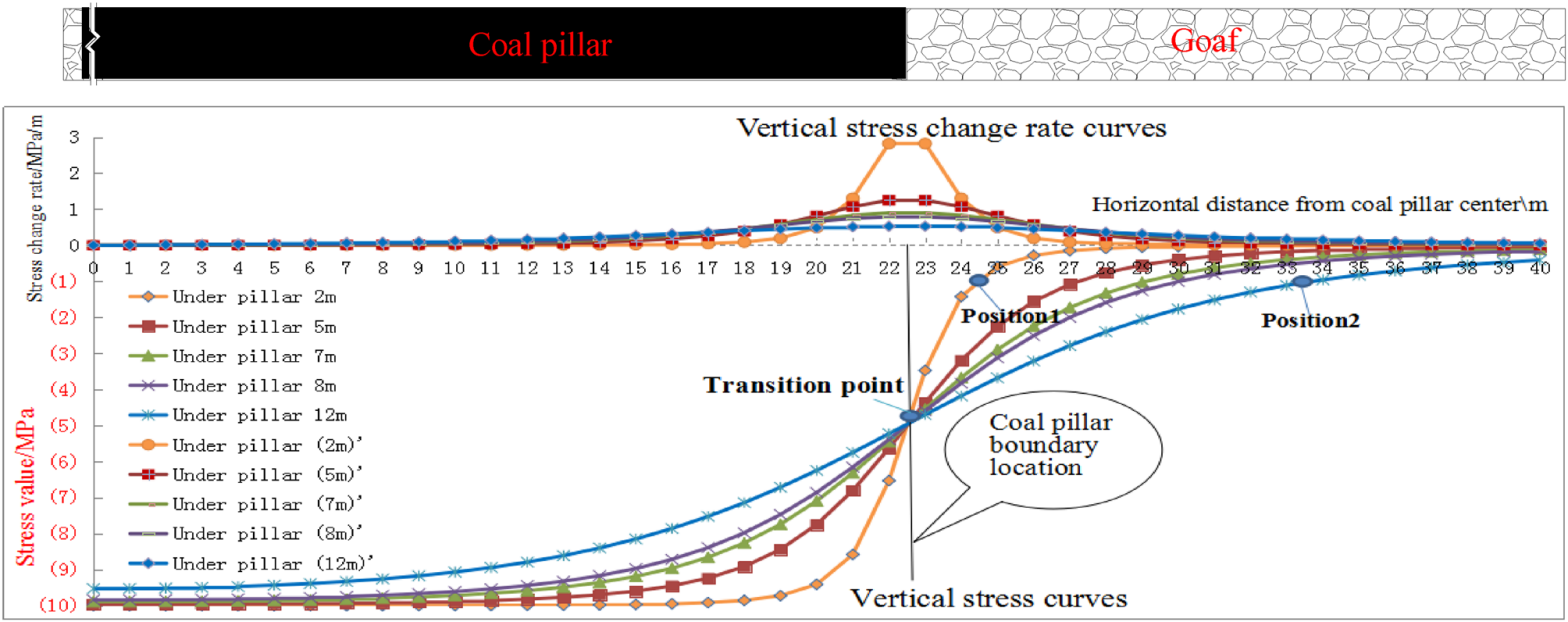
Stress variation curve at different floor depths under coal pillar.

In order to further analyze the law of vertical stress change rate under the conditions of the theoretical calculation formula, it is supposed that the concentrated stress *q* of the coal pillar is − 10 MPa. The position of the coal pillar center is (0, 0) and the range of concentrated stress caused by the coal pillar is 45 m. The vertical stress curve and its change rate curve at different depths (y = 2 m, 5 m, 7 m, 8 m, 12 m) in the floor were drawn at the width direction (y direction)of the coal pillar, as shown in Fig. 8.

The upper part of Fig. 8 shows the vertical stress change rate curves in the floor under the coal pillar at different depths. Under the position of the coal pillar center (y = 0), the vertical stress change rate of the floor at different depths is 0. Under the coal pillar boundary position (y = 22.5 m), the vertical stress change rate of the floor at different depths attains the maximum. With the depth increasing, the vertical stress change rate gradually decreases. In the width direction of the coal pillar, the vertical stress change rate gradually changes from the larger one in the shallow part to larger one in the deep part with the distance from the coal pillar boundary, but the final change rates are both gradually approaching 0.

The lower part of Fig. 8 shows the vertical stress curves in floor with different depths. The following conclusions can be drawn from the analysis of the stress curve.

The stress value of each curve near the coal pillar boundary decreases rapidly. There is a region of intense stress change rate and the stress descending speed decreases with the increase of the depth. Also the range of the stress decline zone keeps expanding with the increase of the depth. For example, in the floor with a depth of 2 m from the coal pillar, the stress value decreases to 1.0 MPa, which is signed at position 1 (1.85 m away from the boundary of the coal pillar). However, in the floor at a depth of 12 m from the coal pillar, the stress value decreases to 1.0 MPa,which is signed at position 2 (11 m away from the coal pillar boundary).

As per the analysis in the previous section, it can be seen that the layout of roadway under coal pillars in close coal seams is not only affected by the magnitude of the concentrated stress, but also by the stress change rate. The roadways should be arranged in areas where the stress changes are small. The vertical stress change rate Eq. (5) in the horizontal direction (y direction) is obtained by the partial derivative calculation in the horizontal direction.

5 $$\frac{{\partial \sigma_{x} }}{\partial y} = \frac{q}{\pi }\left[ {\frac{{2x^{3} }}{{\left[ {x^{2} + (y - l/2)^{2} } \right]^{2} }} - \frac{{2x^{3} }}{{\left[ {x^{2} + (y + l/2)^{2} } \right]^{2} }}} \right]$$

Through the analysis of Eq. (5), the vertical stress in the floor under the coal pillar changes in the horizontal direction of the coal pillar as a binary quartic function of the depth (x) and the horizontal distance (y) away from the coal pillar. By Solving Eq. (6), it’s at the position directly below the center of the coal pillar (y = 0) or the lower boundary interface of the coal pillar (*x* = 0) that the stress change rate is zero, which has no engineering meaning for the selection of the roadway location outside the coal pillars in the close coal seam.

6 $$\frac{q}{\pi }\left[ {\frac{{2x^{3} }}{{\left[ {x^{2} + (y - l/2)^{2} } \right]^{2} }} - \frac{{2x^{3} }}{{\left[ {x^{2} + (y + l/2)^{2} } \right]^{2} }}} \right] = 0$$

According to the influence law of the concentrated stress caused by the coal pillar on the stress distribution of the strata in the floor, the vertical stress and the vertical stress change rate generated by the concentrated stress in the floor outside the coal pillar cannot be zero, but they can be rapidly reduced to a extremely small value. Therefore, it is possible to select the roadway position with lower stress and lower stress change rate near the coal pillar boundary. According to the requirements of the roadway construction in the stress environment, Eq. (7) is used to calculate the horizontal distance between the reasonable roadway position and the center of the coal pillar.

7 $$\left\{ {\begin{array}{*{20}l} {\sigma _{x} = \frac{q}{\pi }\left[ {\arctan \frac{{y - \frac{l}{2}}}{x} - \arctan \frac{{y + \frac{l}{2}}}{x} + \frac{{x\left( {y - \frac{l}{2}} \right)}}{{x^{2} + (y - l/2)^{2} }} - \frac{{x\left( {y + \frac{l}{2}} \right)}}{{x^{2} + \left( {y + \frac{l}{2}} \right)^{2} }}} \right] \le k_{1} q} \\ {\frac{{\partial \sigma _{x} }}{{\partial y}} = \frac{q}{\pi }\left[ {\frac{{2x^{3} }}{{\left[ {x^{2} + (y - l/2)^{2} } \right]^{2} }} - \frac{{2x^{3} }}{{\left[ {x^{2} + (y + l/2)^{2} } \right]^{2} }}} \right] \le k_{2} q} \\ \end{array} } \right.$$

In Eq. (7), *k*_1_ is the stress concentration coefficient and *k*_2_ is the stress change rate coefficient. After simplification, another expression of Eq. (7) can be obtained.

8 $$\left\{ {\begin{array}{*{20}l} {\left[ {\arctan \frac{{y - \frac{l}{2}}}{x} - \arctan \frac{{y + \frac{l}{2}}}{x} + \frac{{x\left( {y - \frac{l}{2}} \right)}}{{x^{2} + (y - l/2)^{2} }} - \frac{{x\left( {y + \frac{l}{2}} \right)}}{{x^{2} + \left( {y + \frac{l}{2}} \right)^{2} }}} \right] \le \pi {\text{k}}_{1} } \\ {\left[ {\frac{{2x^{3} }}{{\left[ {x^{2} + (y - l/2)^{2} } \right]^{2} }} - \frac{{2x^{3} }}{{\left[ {x^{2} + (y + l/2)^{2} } \right]^{2} }}} \right] \le \pi k_{2} } \\ \end{array} } \right.$$

Through the analysis of formula (8), the reasonable horizontal distance y between the roadway and the coal pillar center is an unary quartic function of the vertical distance x between the roadway and the coal pillar. The selection of the horizontal position of the roadway in coal or rock strata with specific depth under the coal pillar depends on the stress attenuation coefficient *k*_1_ and stress change rate coefficient *k*_2_. However, the concentrated stress intensity *q* generated by the coal pillar determines the specific size of the stress value and the stress change rate value at the location. Therefore, the specific coefficients *k*_1_ and *k*_2_ should be selected according to the roadway support strength and the self-supporting capacity of the roadway surrounding rock.

## Analysis on roadway location selection influenced by coal pillar

### Case analysis

**Figure 9:**
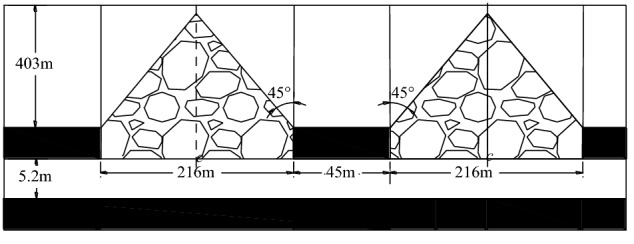
Schematic diagram of coal pillar load calculation.

According to the actual situation of the roadway project in the test Mine, the overburden depth *H* of the #4 coal seam pillar is 403 m, and the width *l* of coal pillar is 45 m. The length *D* of the goaf on both sides of the coal pillar is 216 m. The average bulk density *γ* of coal pillar overburden is 25KN/m. The breakdown angle of the roof on both sides of coal pillar is 45º. According to the method of Ultimate Strength Approach^12,31^(Fig. 9) and those parameters mentioned above, the load *q* of coal pillar is 52 MPa. According to the Three-Index Method^23^, when the stress concentration coefficient and stress gradient are both less than 1, the roadway surrounding rock are easier to control. In the engineering application, the critical position where the stress value is less than the original rock stress and the change rate of stress in the horizontal direction is less than 1 MPa/m is the roadway critical position. The roadway is arranged in #5 coal seam under the coal pillar in #4 coal seam. The vertical distance between the roadway and the coal pillar is 5.2 m. According to Eq. (7) and the stress environment requirements of the critical position, it is determined that the critical position is 29 m from the center of the coal pillar. In other words, the horizontal distance from the coal pillar boundary is 6.5 m, which meets the engineering requirements.

**Figure 10:**
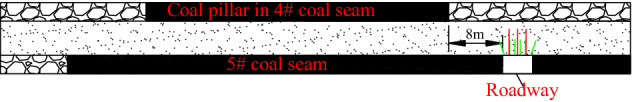
The location and support of roadway.

According to the numerical fitting equation in Fig. 6, the optimal horizontal position of the roadway is calculated as 6.36 m away from the coal pillar boundary, which is similar to the result calculated by the theoretical formula. According to the comprehensive calculation results, the reasonable roadway position under the residual coal pillar of the #4 coal seam is more than 6.5 m away from the coal pillar boundary. Considering the variation of coal seam spacing and the existence of the plastic zone in the roadway surrounding rock, the horizontal distance between the roadway in #5 coal seam and the coal pillar boundary of #4 coal seam is determined as 8 m (Fig. 10). The roadway adopts “anchor + net + cable” support form.

**Figure 11:**
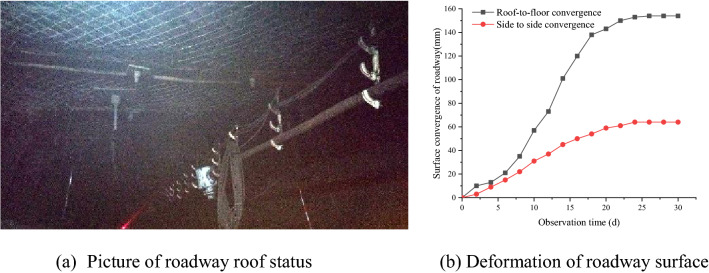
Deformation observation of roadway surrounding rock at construction site.

Mine pressure observation of the roadway was performed on site, and turned out the roadway mining pressure was not obvious. The overall deformation of the roadway was very small, and there was no layer separation in the roadway roof. Engineering application results that, the roadway has avoided the influence of coal pillar concentrated stress, and a good control of the surrounding rock of the roadway was attained (Fig. 11).

### Physical similarity simulation analysis for engineering applications

**Figure 12:**
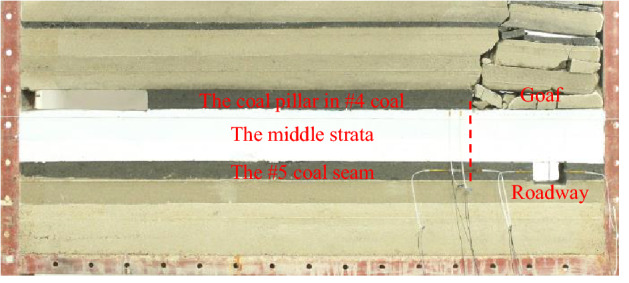
Physical simulation integral model.

To study reflect the propagation law of coal pillar concentrated stress in the floor, a physical similarity model was established as per the engineering practice, and the loading test was performed (Fig. 12).

The occurrence relation and physical and mechanical properties of the simulation strata are consistent with the numerical simulation model. The geometric size ratio of the model is 1:50, and the ratio of similar materials weight refer to the ratio table of similarity tests^32^.

**Figure 13:**
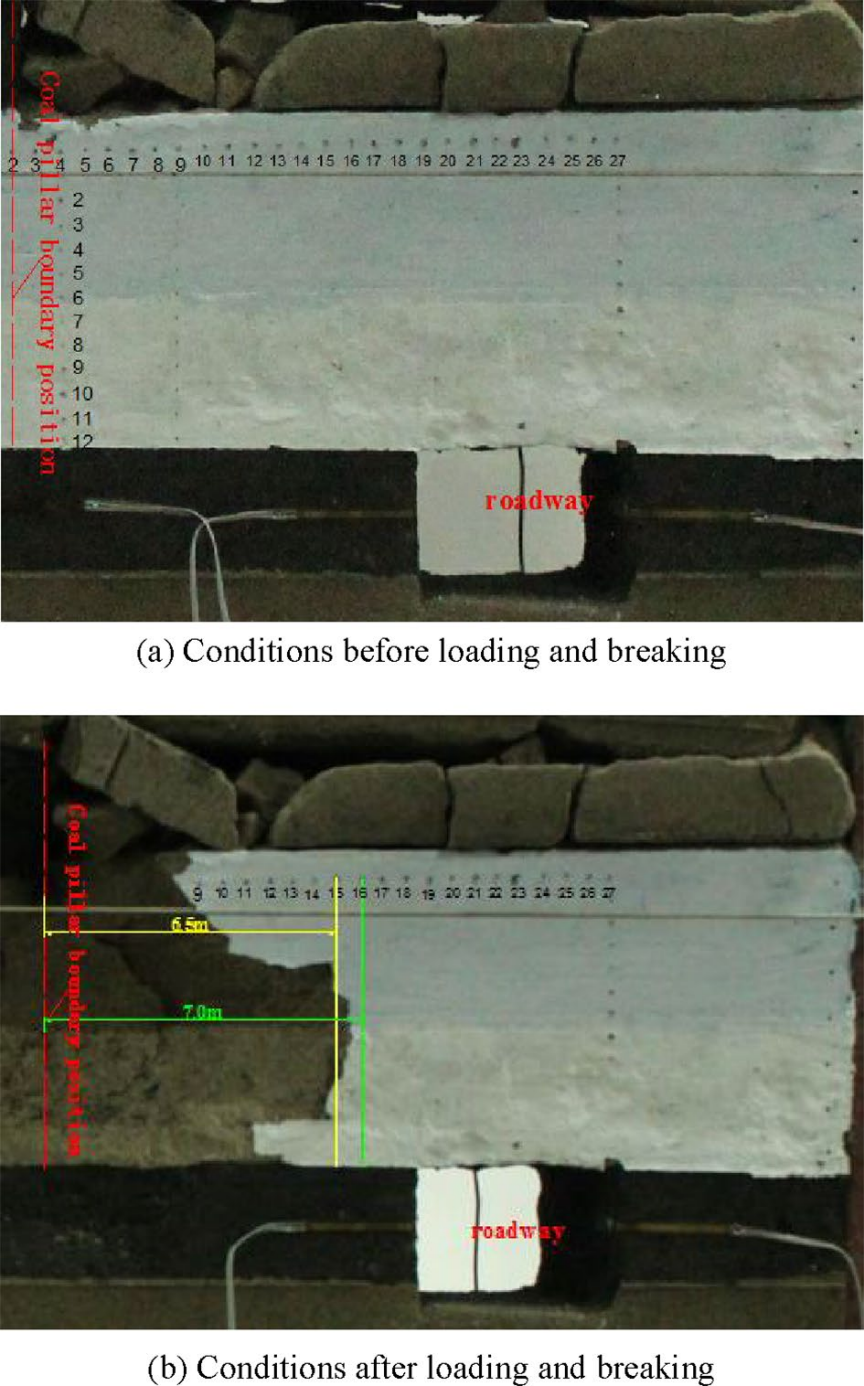
State of coal and rock strata at roadway coal pillar side before or after loading.

Throughout the test, the hydraulic cylinder was used to load the top of the model until the coal stratum under the coal pillar was destroyed. With the loading going on continuously, the coal strata under the coal pillar gradually broke down, and finally the plastic failure occurred in the high stress zone and the stress decreasing zone under the coal pillar. The failure range extended to a horizontal distance of 6.5~7 m from the coal pillar boundary (Fig. 13), which was consistent with the theoretical results. The simulated roadway was 8 m away from the coal pillar boundary and was rarely affected by the concentrated stress of the coal pillar so that the surrounding rock of the roadway was stable.

## Conclusions

Based on the numerical simulation results, the influence of the concentrated stress caused by the coal pillar was gradually weakened, and the stress gradually was transferred to both sides of the coal pillar. The range of stress concentration zone under coal pillar decreases with increasing depth, while the range of stress decreased zone increases with increasing depth. The critical position of the stress decline zone can be obtained by fitting the stress function according to the numerical simulation results, and this position is taken as the horizontal critical position of the roadway layout under the coal pillar. The results provide a solution for selecting roadway layout with similar specific geomechanical conditions.

According to theoretical analysis of the stress propagation, the stress curve of different depth positions was calculated and the stress propagation characteristics were evaluated. The joint formula of stress and stress change rate were established to determine the appropriate roadway position meeting the engineering requirements, which provides a mathematical method for selecting the horizontal position of roadway under the coal pillar.

Engineering application was performed in a typical coal mine based on previous analysis. The horizontal distance between coal pillar boundary and the roadway in the close coal seam under the coal pillar was calculated by using the fitting function of numerical simulation and the elastic-based calculation formula. The reasonable roadway location significantly improved the roadway surrounding support effect. Physical similarity simulation test verifies that the stress transfer range and plastic failure range are outside the roadway layout position. Therefore, the rationality of the roadway location calculation result is further verified.

## Data Availability

The datasets used and analysed during the current study available from the corresponding author on reasonable request.
